# Supplementary material for: Phenotypic and Genomic Characterization of Streptomyces pakalii sp. nov., a Novel Species with Anti-Biofilm and Anti-Quorum Sensing Activity in ESKAPE Bacteria
Source: Microorganisms. 2023 Oct 13;11(10):2551. doi: 10.3390/microorganisms11102551 (PMC10608816; doi:10.3390/microorganisms11102551)
Supplement: Supplementary file 1 [file microorganisms-11-02551-s001.zip › microorganisms-2635752-supplementary/MS MDPI/Suplementary material.pdf]

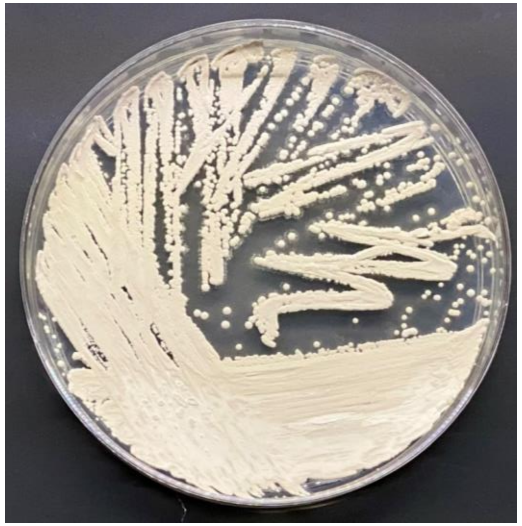

Control (GAE medium)
28°C / 7 days

ISP2 **ISP3** **ISP4**

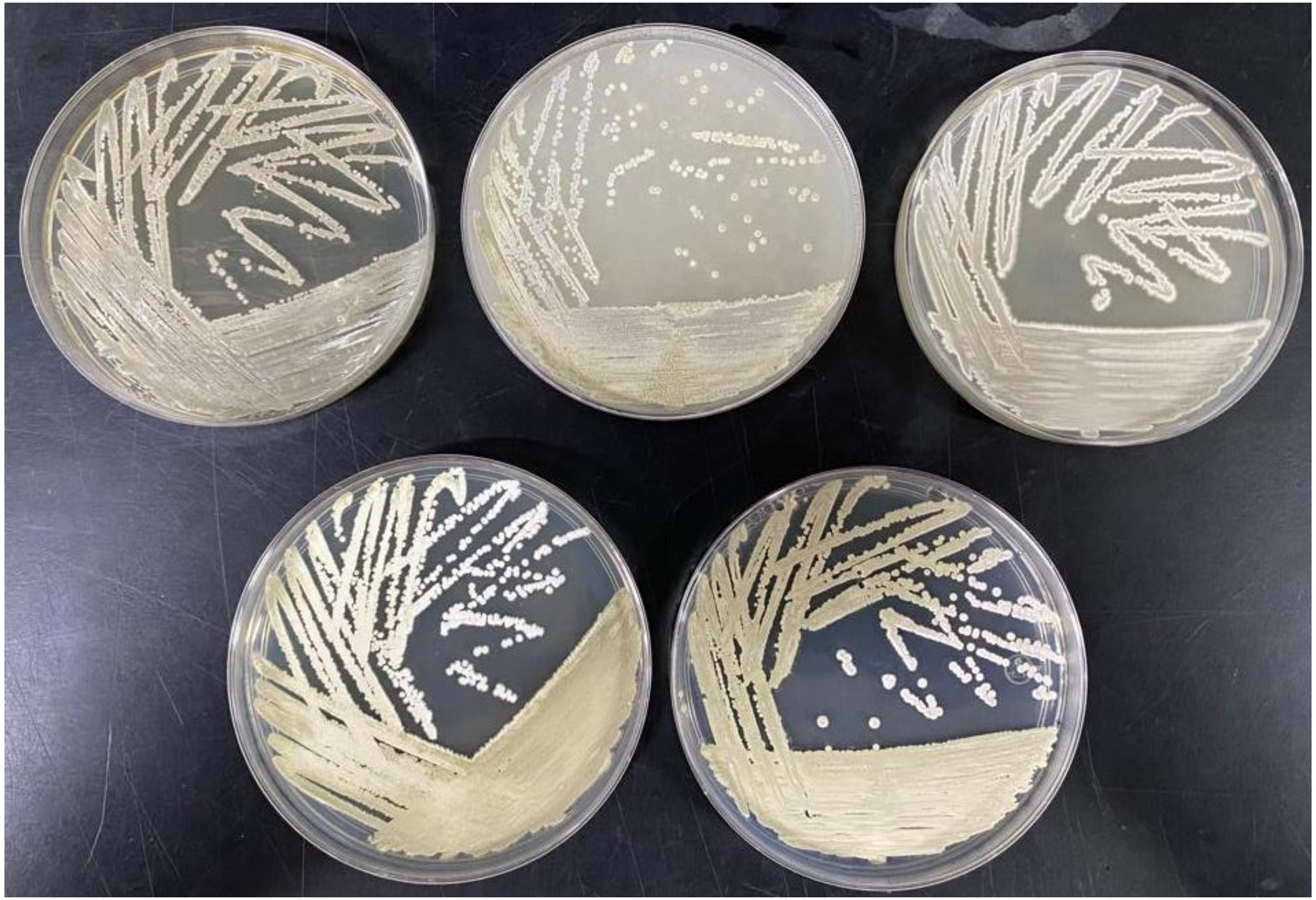

ISP5 **ISP7**

Supplementary Figure S1.

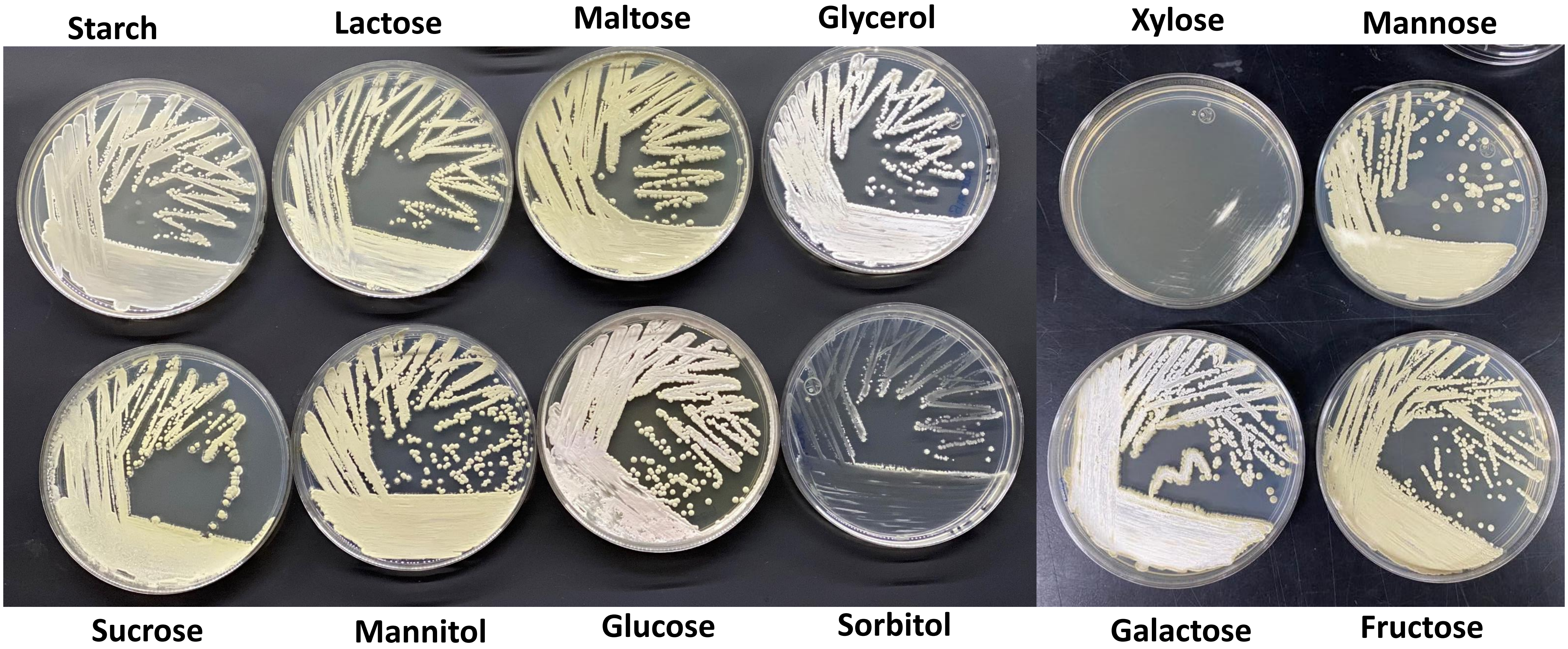

Supplementary Figure S2.

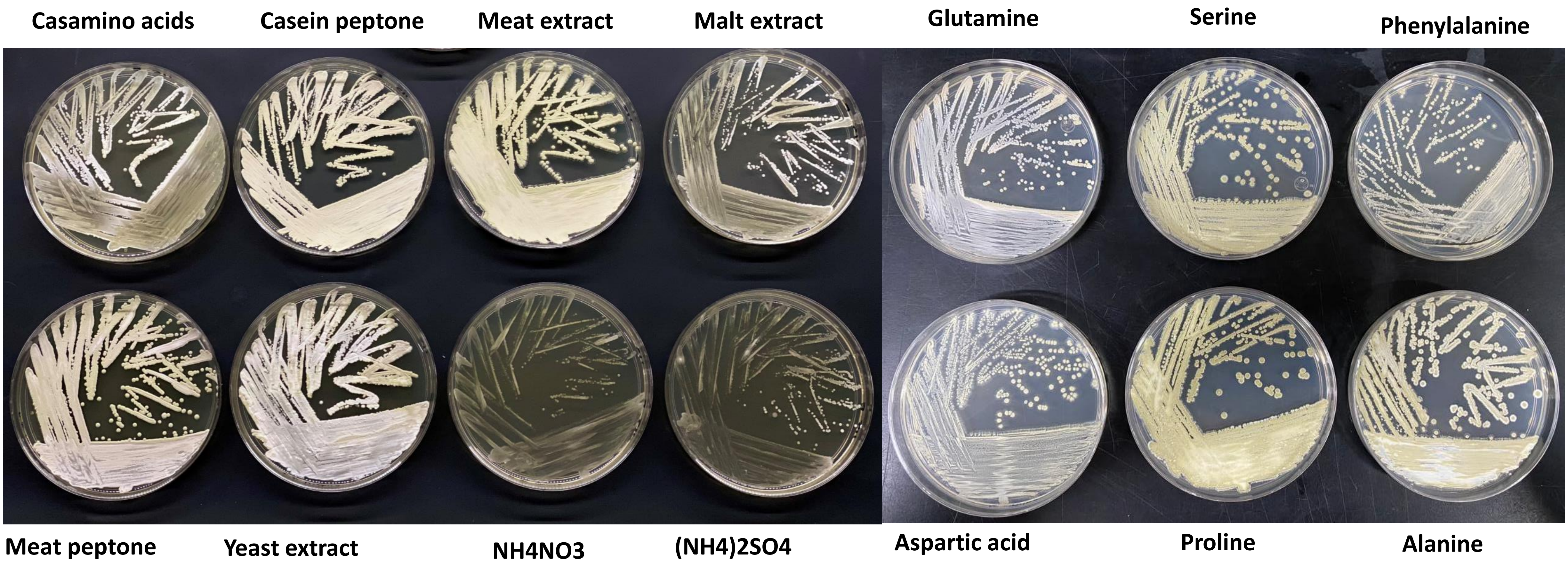

Supplementary Figure S3.

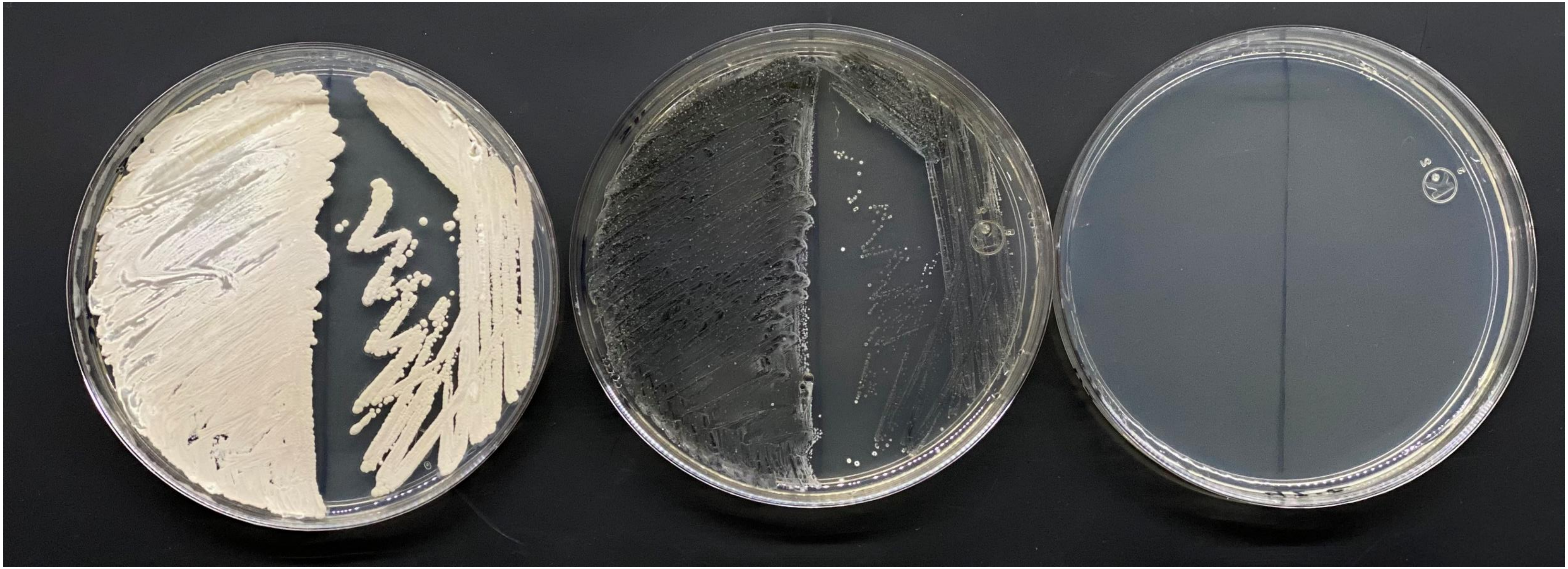

Incubation temperature: 28°C 37°C 42°C

GAE medium / 7 days

Supplementary Figure S4.

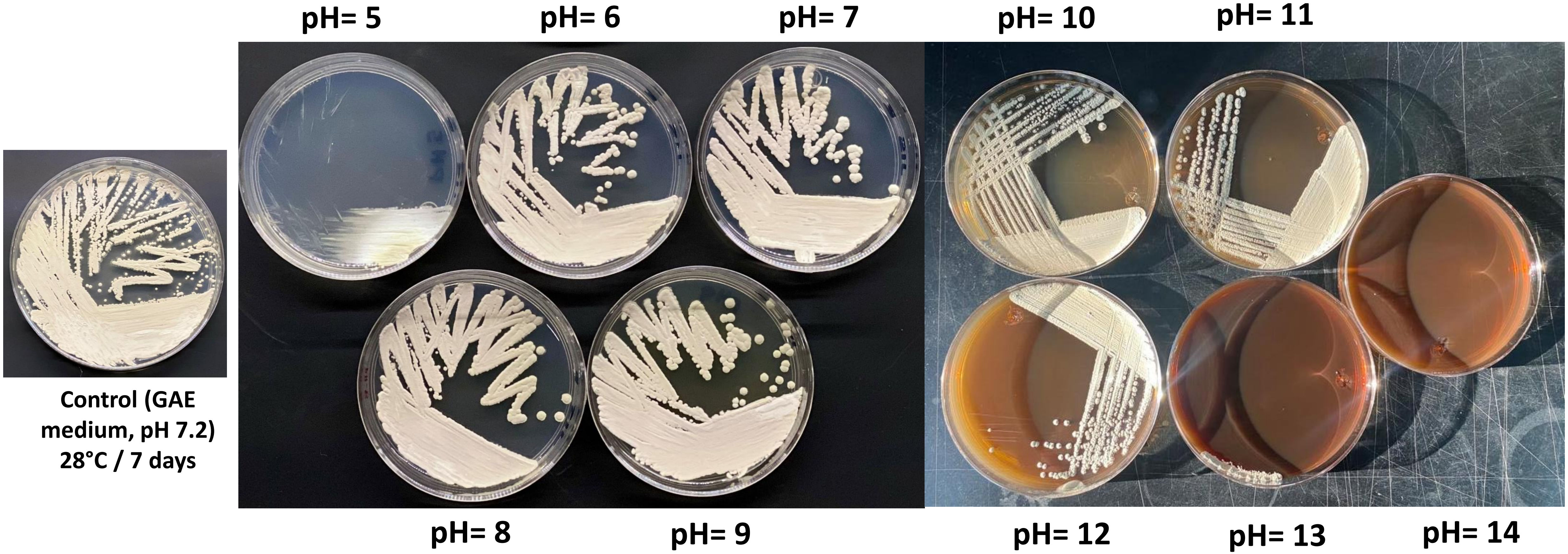

Supplementary Figure S5.

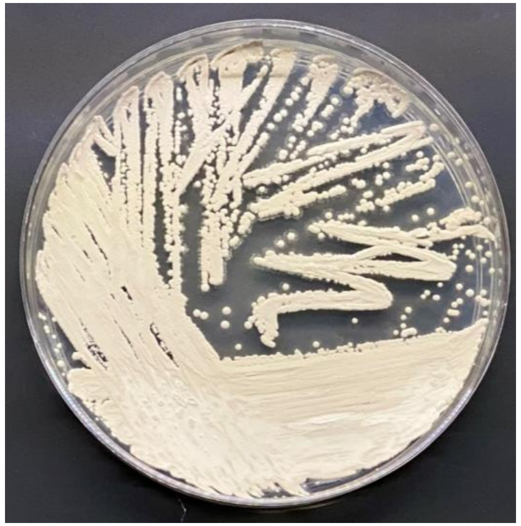

Control (GAE medium, 0% NaCl)
28°C / 7 days

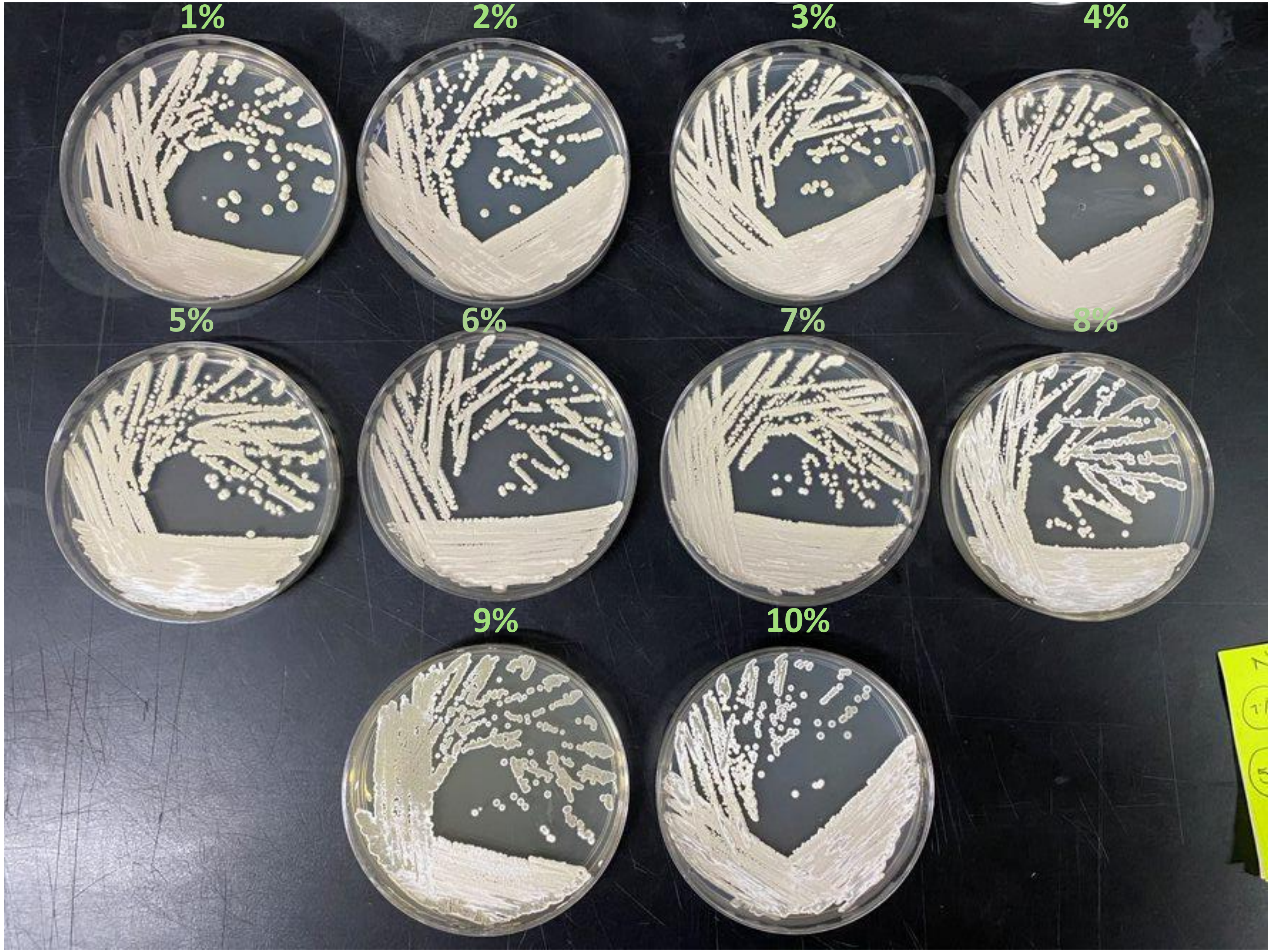

NaCl concentration

Supplementary Figure S6.

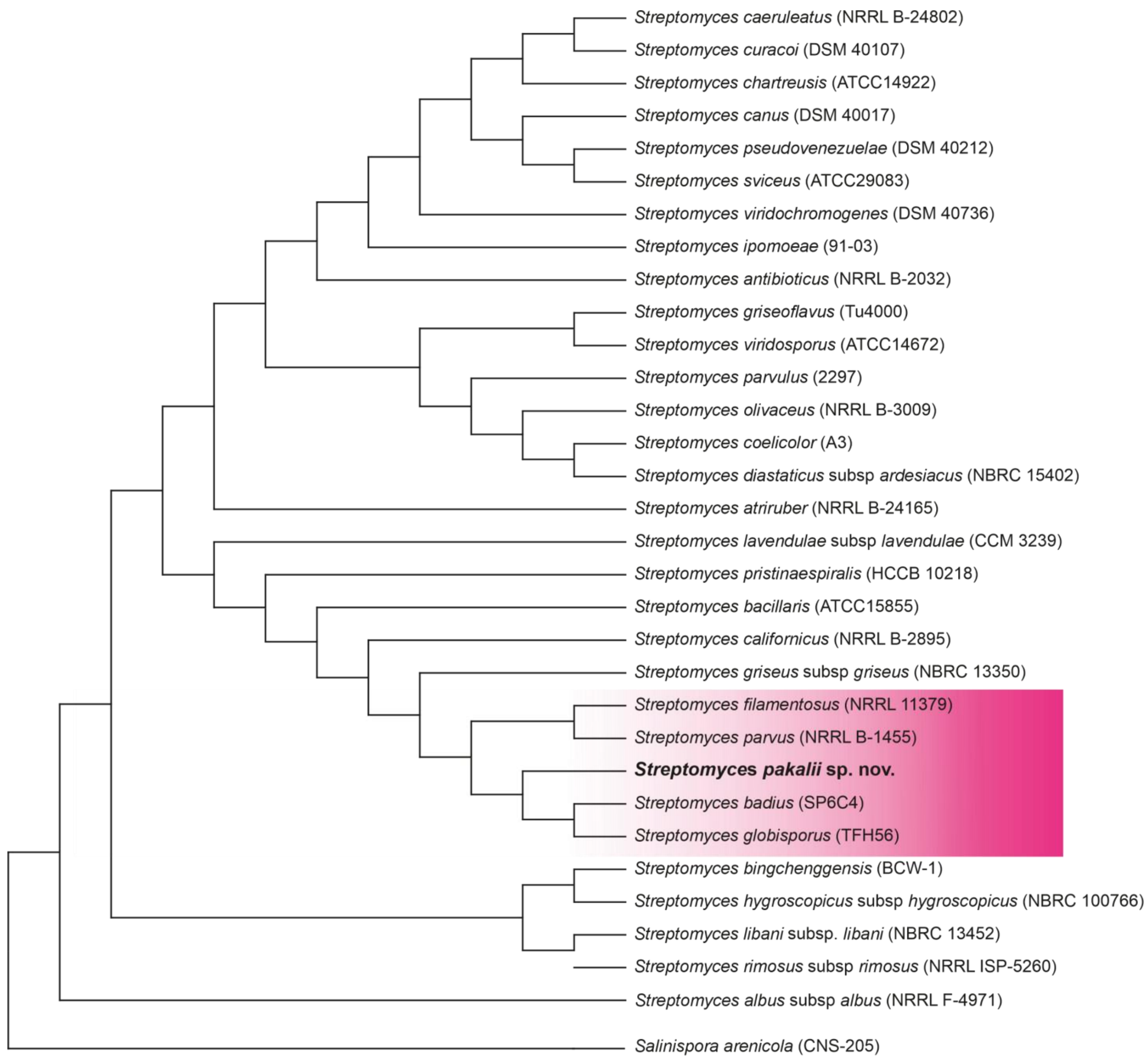

Supplementary Figure S7.

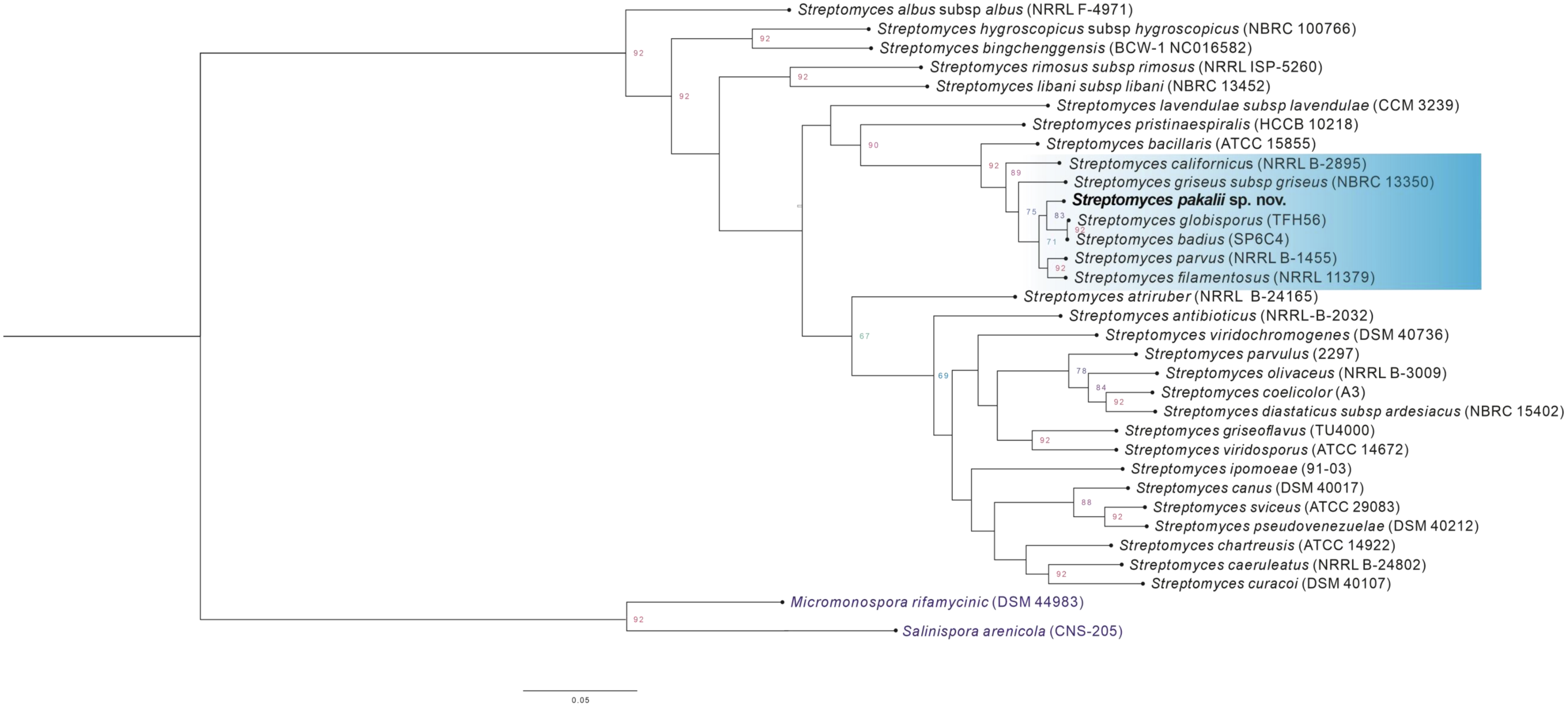

Supplementary Figure S8.
